# Guinea Worm Eradication: New Clinical and Public Health Challenges at the Last Mile

**DOI:** 10.3390/tropicalmed11080218

**Published:** 2026-08-04

**Authors:** Barry R. Davis, John Dunn, Lisette V. Alcantara, Dennis G. Maki, Timothy D. Dye, Charles H. Hennekens

**Affiliations:** 1 Biostatistics and Data Science, Center at the University of Texas-Houston, School of Public Health, University of Texas Health Science, Houston, TX 77030, USA; barry.r.davis1@gmail.com; 2 Department of Population Health, Charles E. Schmidt College of Medicine, Florida Atlantic University, Boca Raton, FL 33431, USA; lalcantara@health.fau.edu (L.V.A.); dyet@health.fau.edu (T.D.D.); profchhmd@prodigy.net (C.H.H.); 3 School of Medicine and Public Health, University of Wisconsin, Madison, WI 53705, USA; maki.dennis@yahoo.com

**Keywords:** dracunculiasis, eradication, guinea worm, neglected tropical diseases, prevention, public health, surveillance, animal reservoirs

## Abstract

Guinea worm disease is approaching eradication, with only 10 human cases reported worldwide in 2025, the lowest annual total recorded. Yet the final phase of eradication presents complex clinical and public health challenges that differ from earlier stages of control. Persistent animal infections, particularly in dogs, civil conflict, fragile health systems, financing disruptions, and the need for prolonged surveillance threaten progress at the point when global attention may wane. Unlike smallpox, Guinea worm has been driven to near-eradication without a vaccine, curative drug, or rapid diagnostic test, relying instead on safe water, filtration, vector control, case containment, community education, reporting incentives, and sustained field operations. This Perspective argues that eradication remains achievable but requires a specific policy agenda: protected financing for surveillance, animal-source transmission control, safe-water measures, operational access, and certification through 2030. Completing the final mile would represent a permanent preventive achievement and a durable victory for neglected populations too often overlooked.

## 1. Introduction

Guinea worm disease is a parasitic infection caused by *Dracunculus medinensis* [1,2]. Humans become infected by drinking water containing infected copepods and, in some settings, by eating inadequately cooked aquatic animals or raw fish entrails [1,2,3]. About a year later, the mature female worm migrates to the skin, usually in a lower extremity, producing a painful blister through which the worm emerges [1,2]. The disease is rarely fatal, but often causes significant disability, leaving affected individuals unable to walk or work for weeks or even months [1,2]. In endemic communities, the disease has historically reduced agricultural productivity and increased school absenteeism [1,4].

Few public health successes in disease prevention are as remarkable as the global campaign against Guinea worm. Modern eradication efforts began in 1980 with an initiative launched by the United States (US) Centers for Disease Control and Prevention (CDC). These efforts later became part of the initiative of the World Health Organization to eradicate *dracunculiasis* [2,5]. Since 1986, the Guinea Worm Eradication Program has been led operationally by The Carter Center in collaboration with ministries of health, WHO, United Nations International Children’s Emergency Fund (UNICEF), CDC, and other partners [4,5]. This governance structure is distinctive because the disease persists in remote, poor, and politically fragile settings, but the campaign has been sustained through an alliance of national governments, multilateral agencies, donors, and a nongovernmental organization that has taken an unusual leading coordinating role [4,5].

The scale of that achievement is difficult to overstate. In 1986, an estimated 3.5 million human cases occurred annually in 20 African and Asian countries. By 2025, only 10 human cases were reported worldwide [4,6]. This decline of more than 99.99% was achieved not through a single biomedical breakthrough, but through sustained application of familiar public health measures: safe water, filtration, larviciding of unsafe water sources, health education, case containment, active surveillance, and community reporting [1,2,4,5].

The efforts of both public health practitioners and researchers are necessary. Researchers need to conduct surveillance to prevent recurrent disease, to understand changing transmission patterns, and to monitor for the potential development of resistance to the current highly effective and essential water larvicide.

This unconventional global structure to achieve eradication is also significant because Guinea worm does not fit the usual model of disease that generally commands sustainable global attention and priority over time. There is no vaccine to deploy, no curative drug to market, and no diagnostic test to anchor a technological solution [1,2,4]. Moreover, the disease offers little commercial incentive and primarily affects populations with the least geopolitical visibility [1,4]. In that sense, Guinea worm has long been susceptible to neglect [1,4]. Its near-eradication is therefore a programmatic achievement as well as a governance success. These successful coordinated efforts demonstrate that global health can, at times, sustain attention to a disease precisely because it is neglected, disabling, and concentrated among disadvantaged communities [4].

The final phase, however, is not merely a smaller version of earlier control work. As human cases have approached zero, the campaign has had to focus increasingly on the possibility that transmission can persist in animals, in unsafe water sources, or in communities where insecurity or displacement limits surveillance [3,5,7]. In eradication, a very low incidence can be misleading. The endpoint is not a dramatic reduction in human disease but the permanent interruption of transmission everywhere, followed by the surveillance and documentation required for certification [7].

## 2. Search Strategy and Selection Criteria

References for this Perspective were identified through searches of PubMed, the WHO website, CDC/MMWR reports, and The Carter Center website for articles and reports published up to July 2, 2026. Search terms included “Guinea worm,” “*dracunculiasis*,” “eradication,” “*Dracunculus medinensis*,” “animal infections,” “Chad,” “surveillance,” “certification,” “tethering,” “temephos,” and “neglected tropical diseases.” Relevant English-language reports, surveillance updates, recent intervention and modeling studies, and policy documents were selected based on relevance to Guinea worm eradication, late-stage eradication challenges, animal reservoirs, surveillance, certification, and global health financing. Priority was given to references from 2023 through 2026 because the late-stage epidemiology, certification requirements, and financing environment have changed substantially in recent years. References from the same institutional source were consolidated where a single current source could support related points. Distinct institutional reports were retained only when they addressed separate clinical, surveillance, certification, financing, or policy questions.

This Perspective is not a systematic review. It is an interpretive synthesis intended for clinicians, public health practitioners, and policy makers who must understand why the last mile differs from earlier phases of disease control. We therefore emphasize current surveillance data, operational constraints, animal infections, and public health lessons rather than historical narrative alone.

## 3. Guinea Worm Eradication at the Last Mile

The landmark successes in the near-global elimination of Guinea worm deserve particular emphasis, in part due to fundamental differences from smallpox, the only human disease that has been fully eradicated [7]. Smallpox eradication relied on an effective vaccine together with surveillance and containment; Guinea worm, by contrast, has been pushed to the brink without vaccination, curative treatment, or diagnostic tests [1,2,4]. The campaign to eradicate Guinea worm has instead relied on prevention through safe water access, filtration, treatment of unsafe water with temephos, community education, rapid case detection, infection containment, incentives for reporting, and surveillance maintained over years [1,2,4,5].

The basic logic of control remains straightforward but operationally exacting. A person or animal with an emerging worm can contaminate a water source when the affected body part enters the water. Larvae released by the worm are ingested by copepods, and people become infected when they drink water containing infected copepods [1,2]. Control therefore requires safe drinking water, filtration, vector control with temephos where appropriate, rapid identification of suspected cases, and containment before water contamination occurs [1,2,5]. At the community level, programs must teach people not to enter water sources when a worm is emerging, provide wound care and bandaging, and maintain credible incentives for reporting suspected cases [3,5].

The operational details matter because eradication is unforgiving. A program that prevents most transmission may be sufficient for control, but not for eradication. As the number of cases falls, the cost and difficulty of finding each remaining infection often rise. Surveillance must reach remote communities where roads are poor, insecurity may prevent routine field visits, and health workers may have many competing demands [3,8,9]. In such settings, the quality of local knowledge, trust, supervision, and reward awareness can determine whether the last infections are found or missed [3,5].

Guinea worm eradication cannot be achieved with any single intervention targeting a specific vulnerable phase within the natural history of the disease [1,7]. Transmission must be interrupted using multifactorial interventions. These include simultaneous attention to water sources, human behavior, animal infections, food handling, local surveillance, and community reporting [3,5]. We concur with the current guidance of WHO, which advocates control measures that include safe water, filtering, case containment, vector control, management of aquatic animal waste, reporting incentives, and proactive tethering of dogs and cats in high-risk areas [1,7]. The WHO recognizes that Guinea worm is not merely a parasitic infection to be contained but an ecological and social challenge that requires multisystem coordination [1,7].

Animal infections now represent the most important change in the late-stage epidemiology of eradication. Since 2012, environmental contamination from infected animals has posed a new obstacle to eradication [3]. This challenge is not a minor complication, because worms infecting animals have been identified as *D. medinensis*, the same species responsible for human disease [3]. Consequently, eradication requires interruption of transmission in both humans and animals. The public health task has shifted from preventing human exposure through unsafe drinking water alone to interrupting a broader ecological cycle involving people, water sources, domestic animals, aquatic animals, and food handling practices.

Chad illustrates the problem most clearly. Human cases have remained few, but dog infections have been numerous enough to sustain concern about ongoing transmission [3,5]. In 2024, Chad reported nine human cases and 281 animal infections, and during January to June 2025, it reported one human case and 80 animal infections [3]. These animal infections were fewer than in the prior comparable periods, which is encouraging, but they remain epidemiologically important. Even a single infected dog entering or contaminating a water source can prolong transmission in a setting where human case counts alone may suggest that the disease is almost gone.

The response to animal infections has required adaptations that differ from traditional human case containment. Current interventions include tethering infected dogs and cats until worms emerge and the risk of water contamination has passed, proactive tethering of eligible dogs during periods of higher transmission risk, management of fish entrails and aquatic animal waste, health education directed at animal owners, and continued use of temephos in unsafe water sources [1,2,3,10,11]. These interventions are behavioral as well as technical. They depend on whether households are willing and able to tether dogs, whether communities accept restrictions that may interfere with normal animal use, and whether field programs can identify high-risk villages early enough to act.

Recent studies have begun to clarify the value of these interventions. A 2025 analysis of proactive tethering in Chad reported that the timing and selection of dogs can influence expected prevention of infections, supporting a more targeted operational approach [10]. A 2026 study in PLOS Neglected Tropical Diseases found that concurrent therapeutic and behavioral interventions were associated with fewer emerging *D. medinensis* worms in dogs in Chad [11]. Modeling work has also suggested that dog screening may serve as a complementary control tool in Chad, where animal infections can persist even when human cases are rare [12]. These studies do not replace field surveillance, but they help programs decide how to allocate scarce time, personnel, and supplies.

## 4. Public Health Lessons and Persistent Challenges

The near-global elimination of Guinea worm disease offers several broad lessons for individual and public health practitioners. First, actionable and durable commitment is not expressed solely through health ministries, budgets, or international resolutions. Commitment must also include villages, schools, and households, where community understanding and sustained local actions are indispensable for the success of campaigns that rely heavily on prevention rather than treatment [1,4,7].

Surveillance is the backbone of eradication, and its importance increases as incidence declines. When cases are common, a weak surveillance system may still detect enough disease to guide broad control measures. When cases are rare, missed infections can determine whether eradication succeeds or fails. Late-stage Guinea worm surveillance must therefore do more than passively count cases. It must maintain community awareness, encourage rapid reporting, verify suspected human and animal infections, investigate rumors, monitor water sources, and document the absence of transmission in places where the disease was once present [3,5,7].

WHO certification criteria make clear that zero reported cases alone are not enough. A country that has interrupted transmission must demonstrate the absence of indigenous cases through active surveillance over a defined pre-certification period, and certification depends on documentation reviewed by the International Commission for the Certification of *Dracunculiasis* Eradication [7]. The criteria also address *Dracunculus* infections in animals and similar infections of animal origin, reflecting the modern epidemiology of the disease [7]. This is essential because a country cannot be considered free of transmission if animal infections continue to represent a credible source of environmental contamination.

The distinction between absence of reports and absence of transmission is critical. In remote or insecure settings, a year with no reported cases may reflect true interruption, but it may also reflect poor access, weak reward awareness, population displacement, or a breakdown in field supervision. Reporting incentives can strengthen surveillance, but they require continuous reinforcement. If community members do not know that a reward exists, or do not trust that the reward will be paid, suspected cases may not be reported rapidly [3,5].

Second, disease-specific campaigns can have broader public health implications. For example, in South Sudan, the coordinated Guinea worm eradication efforts helped establish active community-based surveillance and intervention delivery across thousands of remote communities. These experiences have broader relevance for service delivery in hard-to-reach settings [9]. Similarly, since 2012, animal infections have hindered progress toward elimination, particularly in Chad, where infected dogs have undermined eradication efforts [3,5]. The most recent progress reports state that civil conflict and insecurity in Mali impede access for public health personnel and threaten the near-term possibility of eradication [3]. WHO has likewise warned that sudden financing shocks are disrupting surveillance and essential health services in many low- and middle-income countries, with external health aid projected to fall by 30% to 40% when compared with 2023 [8]. In such a setting, even a campaign with a sound scientific base can falter operationally [3,8].

The biological feasibility of eradication does not guarantee operational success. Guinea worm persists in settings where routine public health work is difficult. Civil conflict, insecurity, seasonal isolation, displacement, and weak health infrastructure can delay case investigation and prevent field teams from reaching affected villages [3,9]. Mali has been a particular concern because insecurity has impeded access for public health personnel, and commercial movement of dogs has added further complexity [3]. These are not peripheral concerns. In the final phase, an inaccessible village or a disrupted reporting chain can be as consequential as a biological reservoir.

Global health financing has become an additional threat. WHO warned in 2025 that external health aid was projected to fall by 30% to 40% compared with 2023, causing immediate disruptions in health services in low- and middle-income countries, including disease surveillance [8]. Guinea worm eradication is vulnerable to such shocks because the program depends on field supervisors, village volunteers, transport, communications, filters, larvicide, rewards, and rapid investigation. These are not glamorous expenditures, but they are the practical infrastructure of eradication. If they are weakened before certification, decades of progress could be placed at risk [8,9].

## 5. Reasons for Cautious Optimism

Despite these concerns, there are reasons for cautious optimism. The Carter Center reported only 10 human cases worldwide in 2025, the lowest annual total ever recorded [6]. Chad reported four human cases and 147 animal infections in 2025, down substantially from prior peak years of animal transmission [6]. WHO has certified 200 countries, areas, and territories as free of *dracunculiasis* transmission, leaving only a small number of countries still requiring certification or completion of precertification [7,9]. These are clear indicators of a campaign that is steadily succeeding [6,7,9].

Recent progress reports also show why optimism must remain cautious. A 2026 MMWR report documented indigenous transmission in six countries as of June 2025—Angola, Cameroon, Chad, Ethiopia, Mali, and South Sudan [3]. Although The Carter Center subsequently reported only 10 human cases for all of 2025, persistent animal infections and fragile surveillance systems continue to threaten eradication [3,6].

Furthermore, there is historical precedent for success even in the most high-burden settings. South Asia and many countries in Africa, once home to major Guinea worm burdens, have already achieved elimination or certification [7,9]. Those successes were not achieved because Guinea worm was easy to eradicate, but through extended surveillance, coordinated national programs, community participation, and sustained field operations maintained until transmission was truly interrupted [7,9].

At this stage, a reduction in cases is not enough. In disease control, very low numbers may represent success. In eradication, however, they do not [7]. As long as even a few cases remain, the parasite retains an opportunity to continue its life cycle and potentially re-expand in humans, dogs, or other animals [1,7]. That is why successful traversing of that final mile in the worldwide eradication of Guinea worm poses so many clinical and public health challenges. Achieving success in that final mile requires sustained persistence after major gains have already been achieved, at precisely the point where fatigue, instability, and competing priorities all create pressures to shift attention elsewhere [3,5,8]. It is our view that Guinea worm eradication should remain a priority despite the many competing threats of premature mortality in this world. Eradication of Guinea worm would represent one of the few permanent improvements that is achievable in public health. Successful completion of this task would prevent the recurrence of avoidable suffering while eliminating the need for future control efforts [7,8].

Late-stage surveillance is also clinically relevant because the diagnosis of Guinea worm disease usually becomes evident only when the worm emerges. There is no widely deployed rapid diagnostic test that can identify all infections during the long prepatent period [1,6]. This means that programs must rely on community recognition of compatible signs, rapid reporting of suspected emerging worms, and laboratory confirmation when feasible. The long incubation period creates additional difficulty because a detected worm reflects exposure that may have occurred roughly a year earlier. By the time an infection is recognized, the water source, household circumstances, animal movements, and security conditions that produced transmission may have changed.

The program also illustrates why prevention can be difficult to sustain. When prevention succeeds, the outcome is a non-event: a child does not miss school, a farmer does not lose weeks of work, a household does not lose income, and a village does not see a contaminated water source seed new infections. These benefits are real, but they are less visible than a new drug, a dramatic operation, or an emergency response. Guinea worm eradication asks the public health community to value absence as an achievement.

There are also lessons for implementation science. The interventions against Guinea worm are not individually mysterious, but their combined delivery is complex. Programs must match interventions to local ecology, seasonality, water use, dog ownership, fish handling, insecurity, mobility, and trust [3,5,10,11,12]. They must monitor whether interventions are being used, not merely whether they have been distributed. They must adapt when transmission pathways change, like what happened with animal infections. In this sense, Guinea worm eradication is not a simple vertical program. It is a dynamic, community-based system for implementing prevention under difficult conditions.

More broadly, Guinea worm eradication would be a permanent preventive achievement. Its completion would demonstrate that sustained international partnership, national leadership, field epidemiology, and local engagement can carry an eradication campaign through its least visible and most difficult phase, even without a new biomedical technology [3,5,7,8].

The most realistic position is therefore neither complacency nor pessimism. Guinea worm eradication is achievable, but it is not automatic. The final cases and animal infections may be sparse, geographically dispersed, and hard to detect. Financing may be uncertain, and insecurity may block access. Still, the combination of current tools, recent evidence, international experience, and community-based operations appears sufficient if sustained. The decisive question is whether the world will maintain attention after the burden has become small enough to ignore but not small enough to declare victory.

## 6. Policy and Recommendations

The final phase of Guinea worm eradication should be managed as a time-limited closing campaign through 2030, rather than as routine surveillance once case counts have fallen. The priority should be to interrupt transmission in the remaining affected countries, then maintain pre-certification surveillance until absence of indigenous transmission is documented [7,13].

Policymakers should fund five specific last-mile functions. First, every high-risk village should have active surveillance, a trained local reporter, clear reward messaging, and rapid investigation of suspected human or animal infections [2,3,5,7]. Second, villages with recent dog or cat infections should receive targeted animal-control support, including proactive tethering, containment of infected animals, safe disposal of fish entrails, and temephos use where indicated [1,2,3,10,11,12]. Third, communities using unsafe surface water should receive filters, safe-water support, and health education focused on preventing contamination of water sources [1,2]. Fourth, programs should reserve contingency funds for insecure areas, seasonal isolation, flooding, population movement, and animal movement, because these are the settings where infections are most likely to be missed [3,8,9]. Fifth, countries approaching interruption should fund data management, independent verification, and preparation of certification dossiers [7].

The central financing principle should be that resources follow transmission risk, not simply the number of reported human cases. A district with no human cases but infected dogs, poor reward awareness, or inaccessible villages should remain a high priority. National programs should report not only case counts, but also the proportion of high-risk villages under active surveillance, time from rumor to investigation, dog-tethering coverage, safe-water access, temephos use where indicated, and readiness for certification review. Funding should therefore be protected first for active surveillance and rapid investigation. Flexible reserves should then support animal-control responses, safe-water measures, access disruptions, and certification activities, with allocations reviewed annually according to transmission risk and operational access.

## 7. Conclusions

Guinea worm eradication during the final mile now poses a practical test of whether the international community can maintain attention long enough to finish what is nearly completed [5,7,8]. The disease has been driven to the margins by steady, often unglamorous public health work in places where both health systems and political conditions are unstable [3,5]. What remains is the shortest but hardest stretch that must include protecting surveillance, preserving partnerships, and maintaining field operations until transmission stops everywhere [5,7]. If that happens, Guinea worm will stand not only as a victory over one ancient disease, but also as a lasting demonstration that prevention ultimately succeeds even in populations and problems the world too often overlooks [7].

Completing eradication would prevent future suffering and remove the need for continuing control efforts. It would also stand as a permanent global health achievement won through safe water, local knowledge, field epidemiology, and sustained international partnership. Finishing the work for communities with too little visibility would affirm the value of disciplined prevention.

This is why the last mile deserves attention from clinicians as well as public health officials. The remaining cases are few, but each represents both preventable suffering and a potential continuation of transmission. For clinicians, Guinea worm shows that supportive care is sometimes necessary precisely because prevention has failed. For public health practitioners, it underscores that eradication requires continuing effort after success has become nearly invisible.

## Data Availability

No new data were created or analyzed in this study. Data sharing is not applicable to this article.

## References

[1] World Health Organization (2025). Dracunculiasis (Guinea-Worm Disease).

[2] Centers for Disease Control and Prevention (2024). About Guinea Worm.

[3] Hopkins D.R., Weiss A.J., Yerian S., Ortega Y.R., Zhao Y., Eneanya O.A., Cama V.A. (2026). Progress Toward Eradication of Dracunculiasis (Guinea Worm Disease)—Worldwide, January 2024–June 2025. MMWR Morb. Mortal. Wkly. Rep..

[4] The Carter Center (2026). Guinea Worm Disease Eradication Program.

[5] Hopkins D.R., Weiss A.J., Yerian S., Zhao Y., Sapp S.G.H., Cama V.A. (2024). Progress Toward Global Dracunculiasis (Guinea Worm Disease) Eradication, January 2023–June 2024. MMWR Morb. Mortal. Wkly. Rep..

[6] The Carter Center (2026). Guinea Worm Disease Reaches All-Time Low: Only 10 Human Cases Reported in 2025.

[7] World Health Organization (2023). Criteria for the Certification of Dracunculiasis Eradication, 2023 Update.

[8] World Health Organization (2025). WHO Issues Guidance to Address Drastic Global Health Financing Cuts.

[9] World Health Organization (2025). Dracunculiasis Eradication: Global Surveillance Summary, 2024. Wkly. Epidemiol. Rec..

[10] Smalley H., Keskinocak P., Swann J., Delea M.G., Eneanya O.A., Weiss A. (2025). Proactive Tethering to Prevent Guinea Worm Infections among Dogs in Chad: An Analysis of the Impacts of Timing and Dog Selection. Am. J. Trop. Med. Hyg..

[11] Dupper A.C., Cleveland C.A., Haynes E.K., Yabsley M.J., Torres-Velez F., Sidouin M.K., Oaukou P.T., Weiss A.J., Garabed R. (2026). Concurrent Therapeutic and Behavioral Interventions Are Associated with a Reduced Number of Emerging *Dracunculus medinensis* Worms in Dogs in Chad. PLoS Negl. Trop. Dis..

[12] Helikumi M., Mushayabasa S. (2023). Dog Screening as a Novel Complementary Guinea Worm Disease Control Tool to Mitigate Persistence in Chad: A Modeling Study. Parasite Epidemiol. Control.

[13] World Health Organization (2024). Three Central African Countries Commit to Global Eradication of Guinea-Worm Disease.

